# Assessing facilitating conditions and barriers for innovation implementation in Canadian long-term care homes: a research protocol

**DOI:** 10.1186/s43058-022-00312-3

**Published:** 2022-06-11

**Authors:** Marie-Soleil Hardy, Maxime Sasseville, Randa Attieh, Laurie-Ann Bergeron-Drolet, Romina Helena Barony Sanchez, Maria Cecilia Gallani, André Côté, Maude Laberge, Philippe Voyer, Vincent Couture, Marie-Pierre Gagnon

**Affiliations:** 1 Faculty of Nursing, Université Laval, Pavillon Ferdinand-Vandry, 1050, avenue de la Médecine, Quebec, QC G1V 0A6 Canada; 2 Public Health Department, Université de Montréal, 7101 Park Avenue, Montreal, QC H3N 1X9 Canada; 3 Public Health Depatment, Université Laval, Pavillon Ferdinand-Vandry, 1050, avenue de la Médecine, Quebec, QC G1V 0A6 Canada; 4 Faculty of Administration, Université Laval, Pavillon Palasis Prince, 2325 Rue de la Terrasse, Quebec, QC G1V 0A6 Canada

**Keywords:** Implementation science, Long-term care, Organizational readiness, Common measurement, Canada

## Abstract

**Background:**

The COVID-19 pandemic has profoundly affected the health and care of older adults, with particularly negative consequences for those residing in long-term care homes (LTCH) and retirement homes (RH). To inform the implementation of interventions with the most potential for impact, Healthcare Excellence Canada identified six promising practices and policy options that can be introduced to ensure that LTCH and RH are better prepared for potential future outbreaks. A total of 22 implementation science teams (ISTs) were funded to support LTCH and RH across Canada in their implementation of these practices. This study aims to identify the enablers and barriers to the successful implementation of evidence-based practices and the impact of intervention in LTCH and RH across Canada.

**Methods:**

A survey-based longitudinal correlational design will be used. The Organizational Readiness for Knowledge Translation (OR4KT) tool will be used to assess the readiness of LTCH and RH to implement the selected practice. The OR4KT includes 59 questions and takes about 15 min to complete. Five to ten respondents per organization, holding different job positions, will be invited by the ISTs to complete the OR4KT in 91 LTCH or RH across Canada at the beginning of the project (T1) and 6 months after the first measurement (T2).

**Discussion:**

The study will provide a benchmark for assessing the readiness of LTCH and RH to implement evidence-based practices. It will also inform decision-makers about barriers and facilitators that influence the integration of promising practices in these organizations.

**Supplementary Information:**

The online version contains supplementary material available at 10.1186/s43058-022-00312-3.

Contributions to the literature

- This study involves 22 implementation science teams and 91 long-term care homes and retirement homes across Canada.
- The study is one of the first to assess the readiness of long-term care homes and retirement homes for innovation implementation.
- Findings from this study have the potential to inform decision-makers, long-term care homes, and retirement homes about facilitating factors and barriers to implement evidence-based practices.
- Lessons learned will support the rapid implementation of new practices and, in so doing, avoid or mitigate future impacts of the COVID-19 pandemic or another outbreak.

## Background

Implementation science is the study of methods and strategies that facilitate the integration of evidence-based practices in health services in a variety of clinical, organizational, and policy contexts [1]. Translating these practices into policies and procedures is one of the greatest challenges in applied health services and policy research, especially given the diversity of contexts [2]. Implementation science theory suggests that context, implementation, and intervention are interdependent and influence each other [3–5].

Implementation science research draws on practical experience to generate knowledge on how to adapt an innovation to a context in different regions or conditions and promote its adoption by various populations. Although the implementation of evidence-based innovations is essential to deliver high-quality care [6], the evidence suggests that the implementation of new evidence-based practices often fails to apply them in a sustainable manner, leading to varying results [7–9]. Hence, the need, through rigorous and pragmatic scientific methods, to better ascertain what works, for whom, under what circumstances, and what factors contribute to the success or failure of the implementation process [1, 3, 4].

The COVID-19 pandemic had profoundly impacted the health and care of the elderly, with devastating consequences for people residing in long-term care homes (LTCH) and retirement homes (RH) (also referred to as nursing homes or congregate living settings for older adults) in Canada. Canadian health agencies recorded a higher national proportion of COVID-19 deaths among residents in LTCH and in RH than any other country worldwide, with over 80% of total COVID-19 deaths occurring in LTCH and RH during the first wave [10, 11].

Faced with the possibility that future COVID-19 outbreaks will coincide with the influenza season, authorities of LTCH and RH are rapidly implementing interventions to improve their preparedness and mitigate the effects of any future outbreaks. To inform the implementation of interventions with the greatest potential for impact, the Canadian Foundation for Healthcare Improvement and the Canadian Patient Safety Institute—now Healthcare Excellence Canada (HEC)—conducted a rapid environmental scan [12]. The aim was to identify the promising practices and policy options that could be applied to ensure that LTCH and RH are better prepared for potential future outbreaks. To support the rapid implementation of these practices and in so doing avoid or mitigate the impacts of subsequent waves of the COVID-19 pandemic or another outbreak, an initiative called LTC + Acting on pandemic Learning was launched in July 2020. With the goal of layering on a research/implementation science lens to this work, HEC partnered with the Canadian Institute of Health Research (CIHR) and funding partners (Health Research British Columbia, New Brunswick Health Research Foundation, Saskatchewan Health Research Foundation, Centre for Aging + Brain Health Innovation) to launch the Implementation Science Teams: Strengthening Pandemic Preparedness in Long-Term Care funding opportunity in September 2020. Through this, 22 implementation science teams (ISTs) and their LTCH and RH partners were funded to lead projects on the implementation of one or more of the HEC six defined promising practices including preparation, prevention, people in the workforce, pandemic response and surge capacity, plan for COVID-19 and NON-COVID-19 care, and presence of family [12]. Moreover, to enable ISTs and their partners in LTCH and RH to collectively understand the facilitating factors and barriers to successful implementation along with the impacts of the intervention, all funded ISTs are invited to participate in a Common Measurement Framework (CMF) project. One of the 22 ISTs was mandated to manage the CMF project. The CMF team consists of 11 researchers, experts in implementation science and long-term care.

This study aimed to identify the enablers and barriers to the successful implementation and the impact of the intervention in LTCH and RH across Canada. To this end, the readiness to implement evidence-based practices in LTCH and RH will be assessed using the Organizational Readiness for Knowledge Translation (OR4KT) tool.

## Methods/design

### Study design

The study uses a survey-based longitudinal correlational design to identify organizational factors associated with the successful implementation of evidence-based practices in LTCH and RH by assessing their readiness to implement the six promising practices.

### Population and setting

The study population includes employees and managers in all LTCH and RH involved in the 22 IST projects funded through the Implementation Science Teams: Strengthening Pandemic Preparedness in Long-Term Care funding opportunity.

### Outcome measurements

The primary outcome is organizational readiness to apply evidence-based practices using the Organizational Readiness for Knowledge Translation (OR4KT) tool. The tool is based on a solid theoretical foundation and passed through several stages of development and validation, including content evaluation and reliability evaluation, in English, French, and Spanish [13]. The OR4KT is based on existing frameworks to assess organizational readiness to support the implementation of evidence-informed practices [14]. To do so, two systematic reviews, one of organizational readiness theoretical components [15] and another of organizational readiness measurement tools [16], have been carried out. The OR4KT includes 59 items grouped under 6 constructs: organizational climate for change, organizational contextual factors, change content, leadership, organizational support, and motivation [13]. The time to complete the questionnaire is estimated at 15 min. Questions are answered on a 5-point Likert scale ranging from 1 = strongly disagree to 5 = strongly agree. The higher the overall score more prepared organizations are to integrate the selected practices and which LTCH and RH are most likely to successfully implement evidence-based practices [13] (Additional file: OR4KT).

### Recruitment and sampling

A total of 22 ISTs from 18 Canadian academic institutions will be invited to participate in the project. The 22 ISTs, comprising 377 IST members, have partnered with a total of 91 LTCH or RH from all 10 Canadian provinces (none of the ISTs includes homes in the 3 Canadian territories). The 22 ISTs are distributed in two cohorts, as categorized by HEC and CIHR: cohort 1 with 14 teams and cohort 2 with 8 teams. In fact, the delay in recruiting cohort 2 teams for this funding opportunity was due to the late reception of additional funding. The CMF team was introduced by CIHR to cohorts 1 and 2 in January and March 2021, respectively, to explain the purpose and tools of the project.

**Fig. 1:**
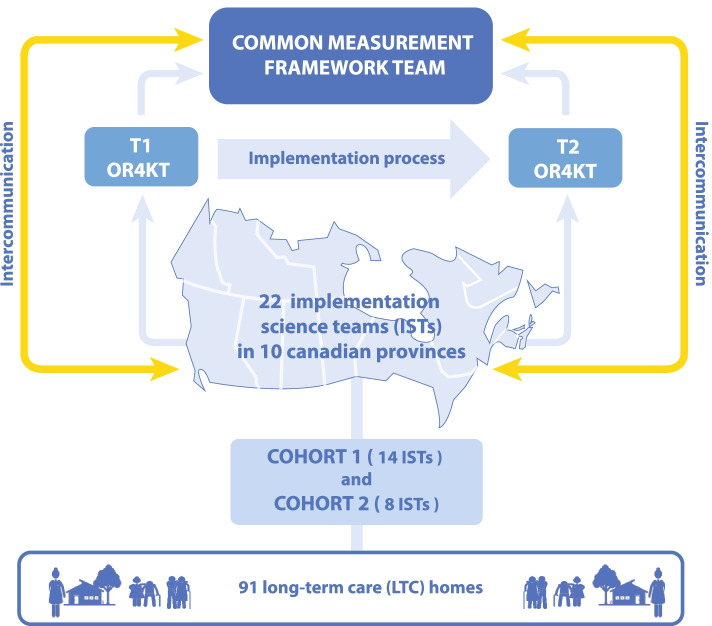
Description of the CMF project progress. This flow chart was elaborated by the CMF team to illustrate the progress of the project

In each LTCH and RH, participants including senior executives (CEO, administrators), middle managers (head nurse, etc.), non-medical staff (housekeeping, food services, etc.), and care workers (health care aids, etc.) will be recruited to participate in the survey. Five to ten participants will be selected from each LTCH and RH by a local implementation science leader for a total of 455 to 910 participants. Given the great variation in size of these LTCH and RH and their staff number, it is not possible to estimate the sample size more accurately. Each IST will contact its affiliated LTCH and RH and decide upon the participation and selection process with respect to respondents (Fig. 1).

### Data collection

The data will be collected in two measurement times T1 (baseline) at the start of the implementation and T2 in post-implementation of the practice, between 2 and 6 months depending on the team. The two data collection times will aim to identify whether changes have occurred in the factors facilitating or constraining the implementation of practices in the LTCH and RH.

We developed a hybrid data collection method to facilitate the collection process by the local implementation science leader, in which questionnaires can be completed on paper or electronically. The questionnaire is available in English and French, the two official languages of Canada. In addition to the 59 OR4KT items, the questionnaire includes four sociodemographic variables for respondents (job title, years of seniority, gender, and age group). Also, descriptive data on LTCH and RH (type of facility, public/private, province, region, number of beds, staff, COVID-19 immunization program) will be provided by senior executives.

The REDCap platform hosted on secure servers at Université Laval will be used for data entry and electronic data collection. For the electronic version, an automatic reminder will be sent to participants if they agreed to participate but have not completed all the surveys. Paper questionnaires will be sent to the Common Measurement Framework (CMF) team by the local leader of the IST by email or scanned and sent electronically. All identifying information will be removed, and all paper questionnaires will be entered manually by a research assistant into the REDCap database and validated by a second research assistant. All data will be stored digitally and backed up on a secure internal server protected by an intrusion detection system and a firewall.

### Data analysis

At baseline (T1), sociodemographic data will be used for descriptive and correlational analyses. First, all survey questionnaires from the same LTCH and RH will be grouped, and a mean score will be computed for each variable. Then, the global OR4KT score and its 5 subscales will be calculated for each LTCH and RH. This information will provide an assessment of the baseline readiness score. In a previous study, an OR4KT score of 64 out of 100 was associated with a high level of readiness to implement new practices in primary care [17]. As there are currently no data on LTCH and RH, we will use this score as a cutoff point to dichotomize the readiness level of LTCH and RH at baseline. We will perform a logistic regression to identify the organizational characteristics associated with the level of readiness of LTCH and RH to implement new practices.

At follow-up (T2), the mean scores and differences in scores (T2 − T1) will be calculated for the overall and subscale scores of the OR4KTfor each LTCH and RH. Considering the baseline OR4KT scores, we will identify which organizational characteristics are associated with a change in the OR4KT scores between T1 and T2 using ANOVA. We will also compare the characteristics of the homes that will show significant improvement, reduction, or no change in the OR4KT score over the 6-month period. We will use a generalized estimating equation model to evaluate the effect of organizational and contextual variables (organization size, organization type, region, vaccination site, etc.) on the OR4KT score differences, a modeling method adapted for longitudinal data [18].

### Ethical considerations

According to articles 2.1 and 2.5 of the Tri-Council Policy Statement [19], the project does not require approval by a research ethics board. The Integrated Health and Social Services Centres de Chaudière-Appalaches research ethics board (REB) analyzed the associated information and documents and confirmed that the project does not involve living human participants or human biological material and is a quality assurance and quality improvement study. The data collection does not include any personally identifiable information from participants. However, a data sharing agreement has been drawn up by Université Laval and the 22 ISTs. The data sharing agreement protects the collection and transfer of data gathered by disclosing parties. It also protects the assessment and analysis of data by the receiving party. Based on the approval and signing of the data sharing agreement by the research ethics board of each IST, the latter agrees to share its data.

### Knowledge translation

We follow the integrated knowledge translation planning guide from the CIHR [20]. At the launch of the project, the complete research plan was presented to the knowledge users, namely the IST members, HEC, and CIHR, during an online meeting. Participants were invited to ask questions to the CMF team. Over the course of the project, we will organize at least two knowledge translation meetings, one at the midterm of the research process and another one at the end of the project. We will integrate all relevant comments and provide any data that is requested, respecting the conditions stated in the data sharing agreement.

To facilitate communication between the CMF team, the 22 ISTs, and between the teams, a group has been created on the collaborative communication platform Slack. In addition, the exchange of information and documents related to the CMF project (protocol, data sharing agreement, questionnaire) was carried out through different communication methods such as emails, Slack, Zoom, and Teams meetings.

The Common Measurement Framework project will also include virtual knowledge exchange activities with the goal of promoting inter-team learning to mobilize experiential knowledge and research results in order to identify common indicators and set priorities. To facilitate these exchanges, a virtual community of practice for all ISTs will be accessible via Slack.

## Discussion

For each LTCH and RH, a global score of organizational readiness to implement evidence-based practices will be determined, as well as scores for the subscale’s organizational climate for change, organizational contextual factors, change content, leadership, organizational support, and motivation. In addition, a list of indicators affecting the implementation of promising practices and the overall impact of these measures in LTCH and RH will be identified and shared in an anonymized report. We will collaborate closely with ISTs to initiate a conversation on how to leverage the facilitating factors and avoid barriers in the implementation of best practice innovation. The collaboration could lead to a tool kit to be used by LTCH and RH and more broadly outside of the IST initiative. The results of this study could inform decision-makers, LTCH, and RH about the facilitating factors and barriers to implement evidence-based practices.

## Supplementary Information

**Additional file 1. **

## Data Availability

OR4KT is available as an additional file.

## Abbreviations

LTCH: Long-term care homes
RH: Retirement homes
COVID-19: Coronavirus disease of 2019
HEC: Healthcare Excellence Canada
CMF: Common Measurement Framework
IST: Implementation science team
OR4KT: Organizational Readiness for Knowledge Translation
REDCap: Research Electronic Data Capture
CIHR: Canadian Institutes of Health Research
