# Supplementary material for: Assessing facilitating conditions and barriers for innovation implementation in Canadian long-term care homes: a research protocol
Source: Implement Sci Commun. 2022 Jun 11;3:61. doi: 10.1186/s43058-022-00312-3 (PMC9187889; doi:10.1186/s43058-022-00312-3)
Supplement: Supplementary file 1 — Additional file 1. [file 43058_2022_312_MOESM1_ESM.zip › PE_mail.pdf]

Dear Julie,

Thank you for your message. Please find attached the requested file – please confirm with the authors via an Author Query that it is correct.

Thank you.

With kind regards,

Alex Verbeek
Project Coordinator Open Access, Production

BMC
Van Godewijckstraat 30
3311 GX Dordrecht, The Netherlands
E: alex.verbeek@springernature.com
www.biomedcentral.com

A pioneer of open access publishing, BMC has an evolving portfolio of high quality peer-reviewed journals including broad interest titles such as *BMC Biology* and *BMC Medicine*, specialist journals such as *Malaria Journal* and *Microbiome*, and the *BMC* series.

At BMC, research is always in progress. We are committed to continual innovation to better support the needs of our communities, ensuring the integrity of the research we publish, and championing the benefits of open research. BMC is part of Springer Nature, giving us greater opportunities to help authors connect and advance discoveries across the world

From: Julie Olano <Julie.Olano@straive.com>
Sent: Saturday, May 28, 2022 1:29 PM
To: Alex Verbeek <alex.verbeek@springernature.com>
Subject: PE Query: 10.1186/s43058-022-00312-3 | 43058_2022_312

[External - Use Caution]

Dear Alex,

Please be informed that additional file: OR4KT was cited in the attached manuscript. However, the corresponding e-file was missing. Could you please check and provide us the missing e-file?

Kind regards,

Julie A. Olano
Project Officer

Straive
Julie.Olano@straive.com
