# Supplementary material for: Assessing facilitating conditions and barriers for innovation implementation in Canadian long-term care homes: a research protocol
Source: Implement Sci Commun. 2022 Jun 11;3:61. doi: 10.1186/s43058-022-00312-3 (PMC9187889; doi:10.1186/s43058-022-00312-3)
Supplement: Supplementary file 1 — Additional file 1. [file 43058_2022_312_MOESM1_ESM.zip › CMF-OR4KT_Eng-BMC.doc]

**Common Measurement Framework Questionnaire**

**Introduction**

The OR4KT questionnaire is used to gather information on certain organizational aspects, particularly ones related to changes that might result from promising practice(s) implemented to reduce the effects of the COVID‑19 pandemic on residents, their loved ones and organizations.

In filling out this questionnaire, you should think of the practice in question that relates to: ❑

(Please select the highest priority practice from the following options: 1 = Preparation; 2 = Prevention; 3 = Pandemic response and surge capacity; 4 = People in the workforce; 5 = Planning for COVID‑19 and non‑COVID‑19 care; 6 = Presence of family)

The term “organization” refers to the long‑term care home (LTC Home), retirement homes (RH) or intermediate resource (IR) where you primarily work.

This questionnaire consists of 59 questions on organizational aspects that could influence the successful implementation of practices. It should take about 15 minutes to complete.

All information you provide will be remain strictly confidential. Individual da ta will not be used for any purpose but will be part of a global analysis of all questionnaire responses.

By agreeing to complete this questionnaire, you consent to participate in this study.

Thank you very much for your cooperation!

| **OR4KT**  On a five-point Likert scale, please assess the degree to which the following statements correspond to your organization:  *1 = Strongly disagree ; 2 = Disagree; 3 = Neutral; 4 = Agree; 5 = Strongly agree; N/A = Not applicable* | | | | | | |
| --- | --- | --- | --- | --- | --- | --- |
|  | 1 | 2 | 3 | 4 | 5 | N/A |
| 1-In your organization, staff work together as a team. |  |  |  |  |  |  |
| 2- In your organization, staff are usually quick to help one another when needed. |  |  |  |  |  |  |
| 3- In your organization, mutual trust among staff is strong. |  |  |  |  |  |  |
|  | 1 | 2 | 3 | 4 | 5 | N/A |
| 4- In your organization, the heavy workload reduces intervention effectiveness. |  |  |  |  |  |  |
| 5- In your organization, staff frustration is common. |  |  |  |  |  |  |
| 6- In your organization, ideas and suggestions from staff get fair consideration by senior leaders. |  |  |  |  |  |  |
| 7- In your organization, the formal communication channels work very well. |  |  |  |  |  |  |
| 8- In your organization, staff members feel free to ask questions and express concerns. |  |  |  |  |  |  |
| 9- In your organization, managers are open to staff ideas for improving change. |  |  |  |  |  |  |
| 10- In your organization, managers encourage trying new and different practices. |  |  |  |  |  |  |
| 11- Your organization considers roles and responsibilities as part of the planning process in relation to specific change initiatives. |  |  |  |  |  |  |
| 12- Your organization has necessary support in terms of budget or financial resources to facilitate change. |  |  |  |  |  |  |
| 13- Your organization has necessary support in terms of training to facilitate change. |  |  |  |  |  |  |
| 14- Your organization has necessary support in terms of facilities and equipment to facilitate change. |  |  |  |  |  |  |
| 15- Your organization has necessary support in terms of staffing numbers to facilitate change. |  |  |  |  |  |  |
| 16- In your organization, managers solicit opinions of clinical staff regarding decisions about patient care. |  |  |  |  |  |  |
|  | 1 | 2 | 3 | 4 | 5 | N/A |
| 17- In your organization, staff members have a sense of personal responsibility for improving patient care and outcomes. |  |  |  |  |  |  |
| 18- In your organization, staff members cooperate to maintain and improve effectiveness of patient care. |  |  |  |  |  |  |
| 19- In your organization, staff members are willing to innovate and/or experiment to improve clinical procedures. |  |  |  |  |  |  |
| 20- In your organization, staff members are receptive to change in clinical processes. |  |  |  |  |  |  |
| 21- In your organization, there is willingness to adjust to changes. |  |  |  |  |  |  |
| 22- In your organization, there is ability to exchange ideas and influence decisions related to delivery of patient care. |  |  |  |  |  |  |
| 23- In your organization, there is flexibility to deal with change. |  |  |  |  |  |  |
| 24- In your organization, people are willing to adjust usual routines in response to what is happening around them. |  |  |  |  |  |  |
| 25- In your organization, typically there is ability to adapt new standards or procedures, even those forced upon us. |  |  |  |  |  |  |
| 26- In your organization, proposed changes have been well accepted by patients prior to implementation. (e.g. in a pilot study) |  |  |  |  |  |  |
| 27- In your organization, proposed changes take into consideration the needs and preferences of patients. |  |  |  |  |  |  |
| 28- In your organization, proposed changes appear to have more advantages than disadvantages for patients. |  |  |  |  |  |  |
| 29- In your organization, proposed changes should be effective, based on current scientific knowledge. |  |  |  |  |  |  |
|  | 1 | 2 | 3 | 4 | 5 | N/A |
| 30- In your organization, managers provide effective management for continuous improvement of patient care. |  |  |  |  |  |  |
| 31- In your organization, managers provide staff members with feedback/data on effects of clinical decisions. |  |  |  |  |  |  |
| 32- In your organization, managers hold staff members accountable for achieving results. |  |  |  |  |  |  |
| 33- In your organization, external stakeholders are involved in the planning process. |  |  |  |  |  |  |
| 34- In your organization, all staff members are usually included in decision-making processes. |  |  |  |  |  |  |
| 35- In your organization, there is an innovation decision maker on key organizational clinical committees. |  |  |  |  |  |  |
| 36- In your organization, there is an innovation decision maker on key organizational administrative committees. |  |  |  |  |  |  |
| 37- In your organization, managers are involved in change process. |  |  |  |  |  |  |
| 38- In your organization, clinicians are involved in the change process. |  |  |  |  |  |  |
| 39- In your organization, administrative and clerical staff are involved in the change process. |  |  |  |  |  |  |
| 40- In your organization, team members provide practical support for new ideas and their application. |  |  |  |  |  |  |
| 41- In your organization, assistance in developing new ideas is readily available. |  |  |  |  |  |  |
| 42- In your organization, team members cooperate in order to help develop and apply new ideas. |  |  |  |  |  |  |
|  | 1 | 2 | 3 | 4 | 5 | N/A |
| 43- In your organization, team members provide and share resources to help in the application of new ideas. |  |  |  |  |  |  |
| 44- In your organization, the progress of change is monitored continuously. |  |  |  |  |  |  |
| 45- In your organization, outcomes are monitored continuously. |  |  |  |  |  |  |
| 46- In your organization, periodic outcome measurements are used to evaluate and improve implementation of planned changes. |  |  |  |  |  |  |
| 47- In your organization, dissemination of performance measures to key stakeholders is used to evaluate and improve implementation of planned changes. |  |  |  |  |  |  |
| 48- In your organization, leaders review results to evaluate and improve planned changes. |  |  |  |  |  |  |
| 49- In your organization, there are formal mechanisms established for obtaining feedback related to the proposed change. |  |  |  |  |  |  |
| 50- In your organization, pressure to make change comes from patients. |  |  |  |  |  |  |
| 51- In your organization, pressure to make change comes from staff members. |  |  |  |  |  |  |
| 52- In your organization, pressure to make change comes from senior leaders. |  |  |  |  |  |  |
| 53- In your organization, pressure to make change comes from board members or overseers. |  |  |  |  |  |  |
| 54- In your organization, pressure to make change comes from funding organizations. |  |  |  |  |  |  |
| 55- In your organization, experience has been gained in implementing change from projects or pilot programs and their evaluation. |  |  |  |  |  |  |
| 56- In your organization, managers are knowledgeable about innovation based on their past experience. |  |  |  |  |  |  |
|  | 1 | 2 | 3 | 4 | 5 | N/A |
| 57- In your organization, knowledge is available about how similar innovations are being used by other organizations. |  |  |  |  |  |  |
| 58- In your organization, senior managers promote change by behaving in a manner consistent with it. |  |  |  |  |  |  |
| 59- In your organization, senior managers define the course of change. |  |  |  |  |  |  |
